# Lord Lister: III. Thirty Momentous Years

**Published:** 1918-02-09

**Authors:** 


### LORD LISTER.

### III.—Thirty Momentous Years.

Lister graduated B.A. at the University of London in 1847, and after his short but sharp attack of madness in 1848 he joined University College Hospital, and began his preliminary medical studies. Here he came under the influence of a number of able and distinguished teachers—Lindley, the botanist; Graham, the chemist; Grant, the comparative anatomist; Ellis, the anatomist; W. B. Carpenter, who was distinguished in several branches of learning; Wharton Jones, characterised by Sir William Jenner as one of the greatest Englishmen that ever lived; and Sharpey, the father of modern physiology. In his later studies he served with Walshe, Jenner, and Erichsen, the two former being great and inspiring physicians. Lister took his M.B. degree and his F.R.C.S. in 1852, and at once began to make original investigations, and it is a sufficient commentary on the contemptible efforts that have recently been made to belittle his abilities that even at the early age of twenty-three, when he had only just emancipated himself from the drudgery of working for examinations, he made anatomical and physiological discoveries sufficiently important to attract the attention of high authorities such as Richard Owen in this country and Kölliker on the Continent.

In 1853, at the age of twenty-six, Lister proposed to complete his studies by spending a month in Edinburgh, where Syme, the greatest surgeon of the day, was at the zenith of his career, and then by visiting the chief clinics on the Continent. He went to Edinburgh, but he went no farther. Syme immediately recognised the promising ability of the young man, made a friend of him, taught him, employed him, made him free of his house, and advanced his interests in every way. Lister was fascinated by Syme's ability and methods, became his house surgeon, started lecturing on his own account with Syme's full approval, married Syme's daughter, was appointed assistant-surgeon to the Royal Infirmary in 1855, and was fairly launched at the age of twenty-eight on the career which was to become so distinguished. Yet we are asked to believe that this young Englishman, who after only two years' residence in Edinburgh was thus promoted over the heads of all his contemporaries and many of his seniors, a young Englishman in Scotland, a man with no local influence but that of his own personality, with no local connections, was a man of the most mediocre ability. The fact is that he was a man of brilliant intellect, and that his intellect was backed by moral qualities that would have ensured his success in any profession, even if his intellect had been less brilliant than it was.

Lister's reputation is so completely identified with his wonderful improvement in the treatment of wounds, and knowledge of physiology and pathology has advanced so immensely since his day, that we are apt to forget his researches in other directions; but at the time they were made they were very important. He it was who discovered what happens in the early stages of inflammation, the fundamental pathological process that lies at the root of both surgery and medicine, and his papers on the subject were considered worthy to be read before the Royal Society of London. He it was who distinguished the contractor and dilator muscles of the iris, the function of the pigment cells in the skin of the frog, and the way this function is exercised, a very important contribution to our knowledge of the activity of cells. He it was who showed that the calibre of the arteries is regulated by the action of the nervous system; and in many other directions he made discoveries that would have been enough to establish his reputation as an observer of the first rank, even if he had died before he had ever treated a wound antiseptically. So much for the asses who are always ready to acquire a temporary notoriety by kicking the carcase of a dead lion.

In 1860, when Lister was only thirty-three, and had been in Edinburgh only seven years, his great ability and the high reputation that he had acquired in Edinburgh distinguished him as the proper successor of Lawrie, the Regius Professor of Surgery in the University of Glasgow. The

☛ Previous articles appeared January 26, p. 351, and February 2, p. 371.

**LORD LISTER**—(*continued*).

appointment was made by the Home Secretary, approved by the Queen, and Lister removed from the City of the Forth to the City of the Clyde. Here his life's work was done. Here was opened to him a wide field for the exercise of his brilliant abilities; and he received this important appointment while he was still in the full vigour of youth, when he was full of energy and enthusiasm, and when he had sufficient knowledge to fill the position capably, and sufficient experience, both of his profession and of the world at large, to give him confidence and enable him to hold his own. He had studied the art of teaching, had had already much experience in it, and was capable not only of successfully imparting knowledge, but, what is far more important in a teacher, of communicating to his pupils his own enthusiasm and inspiring them with his own ideals. It was an ideal appointment, no less for the University than for the Professor. Here he lived for nine busy, eventful, fruitful, and therefore happy years, and here his great reputation was made. At the end of nine years he was appointed, in succession to his father-in-law, Syme, Professor of Clinical Surgery in the University of Edinburgh, where also his researches were continued, his treatment of wounds improved and brought nearer to perfection, and where he received the enthusiastic admiration of large classes of adoring students. After eight more years he became, in 1877, Professor of Clinical Surgery at King's College, London, a move that, though it seemed to promise much, was upon the whole unfortunate and regrettable. By this time his reputation was world-wide. His system was adopted all over the world, and was everywhere successful —everywhere but in London. The metropolitan pride of the London surgeons would not permit them to learn from a professor in a Scotch University, Englishman though he was. The conservative traditions of the profession were in arms against any change; of the few London surgeons who adopted the system, fewer still had had the advantage of being trained by Lister. They did not thoroughly understand his principles; they did not follow his methods faithfully; they did not appreciate the importance of following up the case and attending to the dressing themselves; they did not take the sedulous precautions that Lister took to prevent the contamination of the wound; and their practice was not successful. The spectators were not sufficiently acquainted with Lister's methods to know that these crude and faulty methods were not his. They believed that they were witnessing a demonstration of Lister's methods. They saw that these methods failed, and failed disastrously; and they not unnaturally attributed the failure to Listerism instead of to the departure from Listerism. In Scotland a very large number of general practitioners had passed under Lister's hands and been trained in his methods, and most of those who had not actually been trained by him were sufficiently acquainted with his methods to carry them out intelligently under his direction; but in London there was scarcely one general practitioner to whom the after-care of an operation could be entrusted; and Lister was obliged to do everything himself. If he was unable to do so, or if during his absence his dressings were meddled with by men who knew nothing of his system or of the sedulous care it necessitated, the result was disastrous, and Lister and his system bore the discredit of the disaster. The present writer well remembers the first tentative efforts of celebrated London surgeons to operate with antiseptic precautions, and recognised even at the time how ludicrously imperfect these efforts were. Of course they failed, and the failure was put down, not to the wide departure from Listerism, but to Listerism itself. London was the very last place in the civilised world to adopt the antiseptic system. The London surgeons did not adopt it, did not even give it a trial, until its success throughout the rest of the civilised world compelled them reluctantly to follow; and even after it was thoroughly established and even became the accepted method of practice, some of the older leaders of the surgical profession in London still clung to the old ways. What these old ways were will be described in the next article.

